# Intracellular invasion and survival of *Brucella neotomae*, another possible zoonotic *Brucella* species

**DOI:** 10.1371/journal.pone.0213601

**Published:** 2019-04-03

**Authors:** Steven Grant Waldrop, Nammalwar Sriranganathan

**Affiliations:** Department of Biomedical Sciences and Pathobiology, Virginia-Maryland College of Veterinary Medicine, Virginia Tech, Blacksburg, Virginia, United States of America; Faculty of Science, Ain Shams University (ASU), EGYPT

## Abstract

In 1967, *Brucella neotomae* was first isolated from *Neotoma lepida*, the dessert wood rat, in Utah. With little infection data since its discovery, the zoonotic potential of this *Brucella* species is largely unknown. Recent reports of isolation from human cerebrospinal fluid, along with current literature suggest that *B*. *neotomae* has the ability to infect various hosts and cell types. In this report we extend the knowledge of *B*. *neotomae* ATCC 23459’s intracellular invasion and survival abilities to a variety of cell lines through gentamicin protection assays. Some of the phagocytic and epithelial cell lines from various mammalian species represent characteristics of some cell types that could be encountered by *Brucella* in potential hosts. It was found that *B*. *neotomae* ATCC 23459 exhibits generally lower intracellular bacterial CFUs compared to the mouse-passaged strain of *B*. *neotomae* ATCC 23459, *B*. *suis* 1330, and *B*. *abortus* 2308. Ultimately, these observations provide a small piece of the puzzle in the investigation of the breadth of *B*. *neotomae*’s pathogenic potential.

## Introduction

The genus *Brucella* is composed of Gram-negative, non-sporulating, non-motile cocco-bacilli that lack a capsule from the alpha-proteobacteria group. This group is composed of a variety of bacterial species that are generally associated, but not obligated to specific hosts. The hosts of *Brucella* spp. range widely and include rodents, ungulates, marine mammals, and humans. There are currently 11 accepted species of *Brucella*: *B*. *melitensis*, *B*. *abortus*, *B*. *suis*, *B*. *canis*, *B*. *inopinata*, *B*. *ovis*, *B*. *neotomae*, *B*. *pinnipedialis*, *B*. *ceti*, and *B*. *microti*. Many are considered zoonotic with around 500,000 cases of human brucellosis reported worldwide each year [1].

The bacteria are generally spread through close contact with secretions from infected animals, aerosols, and the consumption of unpasteurized dairy products or undercooked meat. All contribute to a concern for public health [2]. Symptomatic patients exhibit flu-like symptoms for up to four weeks before spontaneous recovery followed by the onset of symptoms again (undulant) [1]. Most infected individuals exhibiting undulant fever recover after 4–12 months, but some develop a chronic infection even after treatment [3]. The disease is rarely fatal, but 2% of all untreated individuals die from complications of brucellosis making it an important disease to monitor [2]. Generally, in non-human mammals brucellosis causes abortion with minimal clinical signs of illness. With each discovery of a new species, our knowledge of this clade continues to evolve and new infection profiles emerge from species and strains once thought to be less pathogenic [4–7]. This is best exhibited through the recent isolation of *B*. *neotomae* from two patient’s cerebrospinal fluid igniting a renewed interest in better understanding the pathogenicity of *B*. *neotomae* [8,9].

*B*. *neotomae* is phenotypically smooth and generally produces small circular colonies on blood agar plates after 24–48 hours of incubation. *B*. *neotomae* does have some biochemical growth characteristics similar to that of the classical virulent species *B*. *melitensis*, *B*. *suis*, and *B*. *abortus* [10, 11]. *B*. *neotomae* also has similar intracellular behavior and a profile of virulence factors similar to that of the other classic species, like a type IV secretion system and an LPS O-side chain among others discussed later [11,12]. This along with murine data and macrophage stimulation transcriptional responses similar to *B*. *melitensis*, suggests that *B*. *neotomae* possesses the ability to behave in a similar manor to the classic virulent species [9,12–16]. Since its discovery over 50 years ago *B*. *neotomae* has only been naturally isolated from *Neotoma lepida* and humans, but it also has been isolated from experimentally infected bison, guinea pigs, rodents, and swine [14,17–21]. It is important to note rodents are generally terminal hosts for *Brucella* spp. [22, 23]. Due to its theorized low pathogenicity, it has been classified as a BSL-2 organism by various entities and investigated as a potential vaccine [8,9,16,19–21]. Attenuation is an important aspect to any vaccine candidate, thus virulence needs further characterization in *B*. *neotomae*. Particularly, it has previously been shown by Gibby and Gibby in 1965 that *B*. *neotomae* develops populations with different infection profiles when passaged in guinea pigs, which could prove troublesome for vaccine work. In our work *B*. *neotomae* ATCC 23459 was passaged through BALB/c mice to produce a stable strain for vaccine specific manipulations. Current literature warrants investigation into the continued non-pathogenic classification and further characterization is needed of *B*. *neotomae* [8,9].

*B*. *neotomae* intracellular survival and growth has been characterized in J774A.1 cells and our study extends this knowledge to a variety of cell lines [8,9,11–13]. Here we use various microbiological assays, like serum opsonization, PCR, and biochemical assays to characterize *B*. *neotomae* ATCC 23459 (BN), a mouse-passaged strain of *B*. *neotomae* ATCC 23459 (BNP2), *B*. *abortus* 2308, and *B*. *suis* 1330. Gentamicin protection assays were used to determine the ability of these species to invade and survive in epithelial and phagocytic cell culture lines from various species and compared to current literature. We hypothesized that BNP2, *B*. *abortus* 2308, and *B*. *suis* 1330 would exhibit higher intracellular invasion and survival patterns compared to BN.

## Material and methods

### Bacterial strains

These strains of bacteria were used: *Brucella neotomae* Stoenner and Lackman 1957 strain 23459 from American Type Culture Collection (ATCC) (Manassas, Va), *B*. *abortus* 2308, and *B*. *suis* 1330 from the culture collection at Virginia Tech (Blacksburg, VA). Bacterial strains were cultured using established protocols and grown at 37 °C under 5.0% CO_2_ atmospheric conditions for 48–72 hours on tryptic soy agar (TSA) (Sigma A-22091) plates [24].

### Passaging of strains through BALB/c mice

All murine experiments were performed in compliance with approved Virginia Tech IACUC guidelines and protocols (IACUC #16–024). One 7-week-old female BALB/c mouse (Envigo) per bacterial strain (*B*. *abortus* 2308, *B*. *neotomae* ATCC 23459, or *B*. *suis* 1330) was inoculated intraperitoneal (IP) with 2.0 x 10^5^ CFUs/mL. Seven days post inoculation the mice were euthanized via carbon dioxide asphyxiation, the spleens aseptically removed, and individually homogenized. Serial dilutions of homogenized spleens were plated on TSA and incubated for 2–3 days at 37°C under 5.0% CO_2_ atmospheric conditions. Single isolated colonies were grown in tryptic soy broth (TSB) and the protocol was repeated once more for each strain. Passaged stock cultures of the above strains were produced following established techniques [15,20]. The original *B*. *neotomae* ATCC 23459 and second passaged strains were used for all of the experiments performed in this paper.

### Identity verification of *Brucella* spp. through presumptive tests

*B*. *neotomae* ATCC 23459, *B*. *neotomae* ATCC 23459 passage 2, *B*. *abortus* 2308, and *B*. *suis* 1330 were used for each presumptive test. Pure bacterial cultures were streaked for isolated colonies, and two to three isolated colonies were used for each presumptive test. One to two drops of oxidase reagent or 3% hydrogen peroxide were directly applied to the isolated colonies and observed for a reaction to occur within 30 seconds [10,18]. An acriflavin (Sigma A-8126) stock solution of 1:1000 wt/v was prepared and used immediately by transferring 2–3 isolated colonies into 30μL of the stock solution on a glass slide. A homogeneous solution was observed for agglutination [10,18]. Thirty microliters of 3-week post-infection mouse serum (VT-COHR sample collection) were mixed with 2–3 isolated colonies on a glass slide and examined for agglutination. A stock solution of 0.1% thionin (Sigma A-861340) and 0.1% basic fuchsin (Sigma A-215597) were prepared in distilled water and autoclaved. TSA was prepared and thionin or basic fuchsin dye (10mL stock dye/1000mL TSA) was added while the liquid medium was still warm. A single isolated colony, 24hrs old, was plated on the TSA plates containing a dye as well as on a TSA plate without dye. The plates were observed for growth after 24-48hrs at 37°C under 5.0% CO_2_ atmospheric conditions [10,18]. The *Brucella* ladder PCR protocol from López-Goñi, I., *et*.*al*. 2008 was followed. All polymerase chain reactions (PCR) were performed using established techniques with Platinum PCR SuperMix High Fidelity (Invitrogen) on a Gradient Mastercycler (Eppendorf) [25]. *Brucella* ladder oligonucleotides primers were purchased from Sigma- Genosys (Sigma-Aldrich, USA).

### Collection of naïve murine peritoneal macrophages

Two 4-month-old female BALB/c mice (Envigo) were euthanized using carbon dioxide asphyxiation. Using a 16 gauge needle 200μL of Dulbecco’s modified Eagle’s medium (DMEM) (Sigma-Aldrich, USA) was injected into the peritoneal space. The medium was retrieved with care to prevent blood from contaminating the samples. The collected peritoneal macrophages were then placed in 50mL Falcon conical tubes. The process of washing the peritoneal space with DMEM was repeated 3 times per mouse. The cells were centrifuged at 3,000 revolutions per minute (rpm) for 5min. The white layer of peritoneal macrophages was resuspended in DMEM supplemented with 2mM ^L-^glutamine, 2mM sodium pyruvate, 10% heat-inactivated fetal bovine serum (FBS), 100 U/mL penicillin, and 100 μg/mL streptomycin (Sigma-Aldrich, USA) and counted under a microscope using a hemacytometer. Wells of a 24-well plate were seeded at 2x10^6^ cells per well with peritoneal macrophages and incubated over night at 37°C under 5% CO_2_ atmospheric conditions before the gentamicin protection assay was performed.

### Opsonization of *Brucella* species

Serum collected from female BALB/c mice 4 weeks post infection with *B*. *neotomae* ATCC 23459 was used to opsonize *B*. *neotomae* ATCC 23459 and *B*. *abortus* 2308 before infection with naïve intraperitoneal murine macrophages. To produce a bacterial suspension for opsonization, a culture of *B*. *neotomae* ATCC 23459 or *B*. *abortus* 2308 in stationary phase was centrifuged for 5min at 3,000 rpm. The bacterial pellet was resuspended in the appropriate cell culture media. The OD_600_ of the cell culture media and bacterial suspensions was determined with a spectrophotometer, and then diluted to an OD_600_ of 0.15 (10^9^ CFUs/mL). Serial dilutions of the cell culture media and bacterial suspensions were plated on TSA plates to determine the CFUs used for each infection. One hundred μL of serum was then added to the suspension and incubated for 30 min shaking at 37°C under 5% CO_2_ atmospheric conditions before the gentamicin protection assays were performed.

### Cellular culture

The cell lines were chosen based on the types of cells that *Brucella* would encounter in the host, with epithelial cells being the initial cell type and phagocytic cells being the ultimate site for replication. The species that the cell lines originate from represent potential zoonotic routes between wild/domestic animals and the public in the United States. Cell lines were cultured as monolayers in T_150_ cell culture flasks (Corning) using aseptic technique and grown at 37°C under 5% CO_2_ atmospheric conditions [13]. The murine monocyte cell line (J774A.1), the bovine monocyte cell line (BM), and the human epithelial cell line (HeLa) were obtained from the culture collection at the Center for One Health Research laboratory building at Virginia Tech. These cell lines were cultured in Dulbecco’s modified Eagle’s medium (DMEM) supplemented with 2mM ^L-^glutamine, 2mM sodium pyruvate, 10% heat-inactivated fetal bovine serum (FBS), 100 U/mL penicillin, and 100 μg/mL streptomycin (Sigma-Aldrich, USA) [26–28]. The bovine epithelial cell line (MAC-T) was obtained from Dr. Isis Kanevsky’s culture collection at Virginia Tech and was cultured in Dulbecco’s modified Eagle’s medium (DMEM) supplemented with 2mM ^L-^glutamine, 2mM sodium pyruvate, 5% heat-inactivated fetal bovine serum (FBS), 100 U/mL penicillin, and 100 μg/mL streptomycin (Sigma-Aldrich, USA) [29]. The human monocyte cell line (THP-1)* and the dog macrophage cell line (DH82) were cultured in RPMI 1640 medium supplemented with 2mM ^L-^glutamine, 10% heat-inactivated fetal bovine serum (FBS), 100 U/mL penicillin, and 100 μg/mL streptomycin (*THP-1 cells were activated/differentiated using 50 ng/mL phorbol myristate acetate (PMA) for 48hrs.) (Sigma-Aldrich, USA), and were also obtained from the culture collection at the Center for One Health Research [30,31]. The swine monocyte cell line (3D4/31) was obtained from Dr. XJ Meng’s culture collection at Virginia Tech and was cultured in RPMI 1640 medium with 2 mM L-glutamine adjusted to contain 1.5 g/L sodium bicarbonate, 4.5 g/L glucose, 10 mM HEPES, 1.0 mM sodium pyruvate, 0.1 mM nonessential amino acids, 10% FBS, 100 U/mL penicillin, and 100 μg/mL streptomycin (Sigma-Aldrich, USA) [32]. The swine epithelial cell line (IPEC-J2) was obtained from Dr. Lijuan Yuan’s culture collection at Virginia Tech and was cultured in DMEM supplemented with 5% FBS, 5μg/ml insulin, 5μg/ml transferrin, 5μg/ml selenium, 5ng/ml epidermal growth factor, 100 U/mL penicillin, and 100 μg/mL streptomycin (Sigma-Aldrich, USA) [33].

### Gentamicin protection assay

Following laboratory protocols for each cell line, the following passage number were used for the gentamicin protection assays: J774A.1—9, DH82—6, THP-1—4, HeLa—5, 3D4/31—5, JPEC-1—3, BM—2, and MAC-T—7. Cell lines were seeded in triplicate per time point/bacterial strain in 24 well plates and incubated overnight in applicable antibiotic free media. The wells were seeded at 2x10^6^ cells per well. After overnight incubation the cells were infected with a multiplicity of infection (MOI) of 1:100 for one hour at 37°C under 5% CO_2_ atmospheric conditions with 48hrs old bacterial cultures at the stationary growth phase in Tryptic soy broth (Sigma-Aldrich, USA). The Log_10_ CFUs/mL of the inoculum can been seen in S1 Table. The bacterial suspensions used were produced as described above. After 1hr of incubation, the infected cells were washed three times with sterile PBS to remove non-phagocytized *Brucella* spp. Each well was filled with 2 mL of media containing 50 μg/mL gentamicin and incubated for 1hr. At specified time points over 48hrs post infection cells were washed three times with sterile PBS and then 200 μL of 0.1% Triton X-100TM (Sigma- Aldrich, USA) lysing agent was added to each well. After 5–10 minutes of incubation in the lysing agent, serial dilutions were plated on TSA plates and incubated for 2 days at 37°C under 5.0% CO_2_ atmospheric conditions to determine intracellular CFUs [13]. For each time point, a portion of the cell culture media was plated on TSA plates to determine if there were free-living bacterial cells in the wells.

### Statistical analysis

All group and time-point data were compared using a One-way ANOVA plus a Dunnett’s correction post hoc test, with both tests significant <0.05. *B*. *neotomae* ATCC 23459 (BN) was used as the species that the other species were compared to. Differences were considered to be significant when *p* values were less than 0.05.

## Results

### Identification and characterization of *B*. *neotomae*

**Fig 1:**
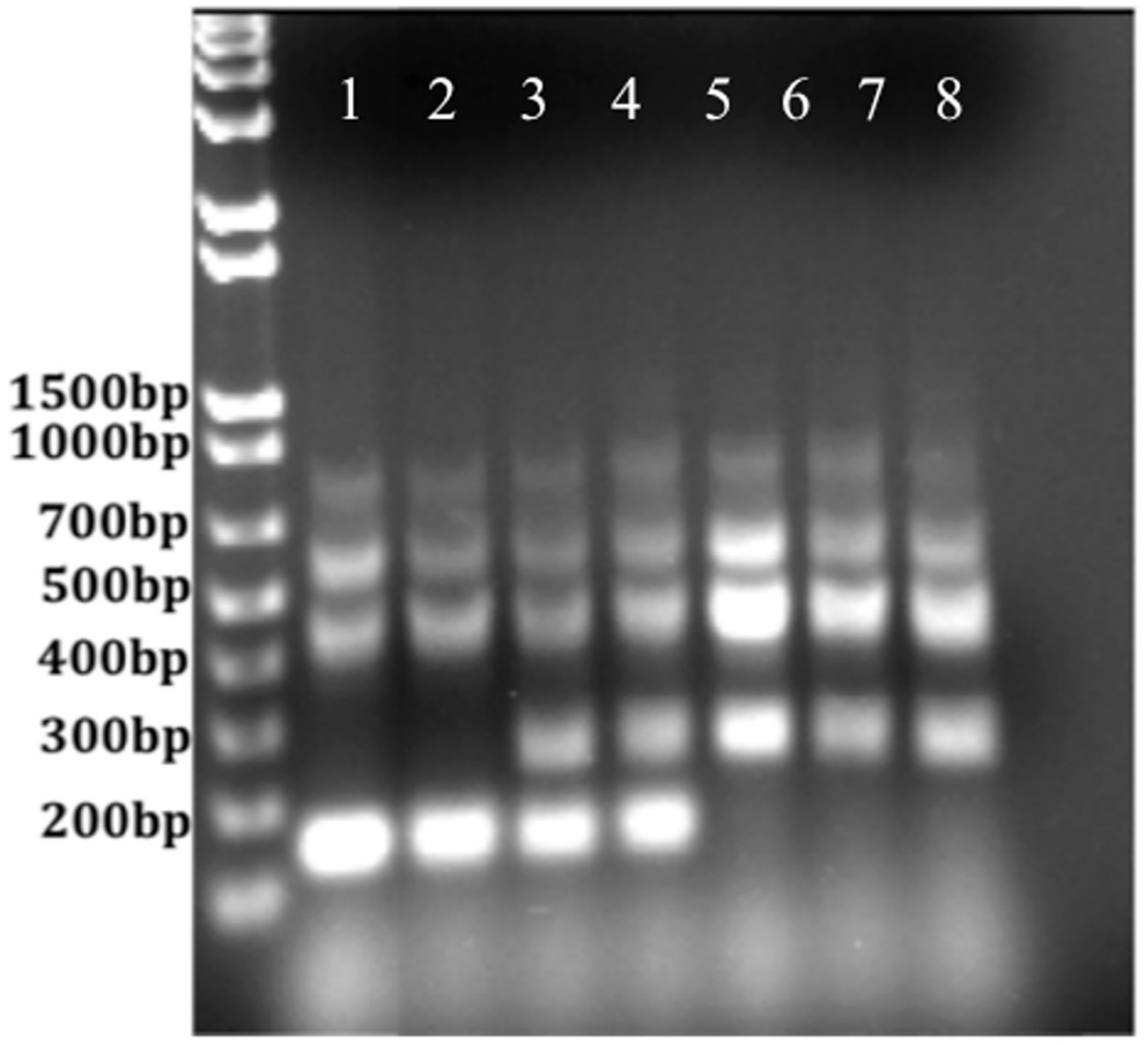
*Brucella* ladder of *Brucella* strains. Represents the *Brucella* ladder PCR banding pattern of the following strains of *Brucella* after the molecular weight marker (1): *B*. *abortus* 2308 passage 1 (2), *B*. *abortus* 2308 passage 2 (3), *B*. *suis* 1330 passage 1 (4), *B*. *suis* 1330 passage 2 (5), *B*. *neotomae* ATCC 23459 (6), *B*. *neotomae* ATCC 23459 passage 1 (7), and *B*. *neotomae* ATCC 23459 passage 2 (8).

*B*. *neotomae* ATCC 23459 (BN) and *B*. *neotomae* ATCC 23459 passage 2 (BNP2) were characterized using several presumptive and biochemical tests. *B*. *abortus* 2308 and *B*. *suis* 1330 were used as control organisms for each test. All tested *Brucella* species grew small circular colonies after 24-48hrs of incubation at 37°C under 5.0% CO_2_ atmospheric conditions. The organisms were Gram negative short rods that exhibited the smooth phenotype when a homogenous suspension was made in a 0.001% solution of acriflavin. The organisms were oxidase negative, catalase positive, grew in the presence of 100 **μ**g/mL of thionin, and did not grow in the presence of 100 **μ**g/mL of basic fuchsin (Table 1). Representative photos of the growth/lack of growth on thionin and basic fuchsin plates can be seen in S1–S3 Figs. Although the *B*. *neotomae* strains exhibited growth on thionin dye that differed from the literature, it has been shown that *Brucella* strains can have some variability in regards to biochemical assay results [20,34]. The organisms still maintained a *Brucella* ladder PCR banding pattern similar to *B*. *neotomae* in the literature (Fig 1) [25]. After passage through BALB/c mice the splenic bacterial CFUs were: 6.96 (*B*. *neotomae*), 6.58 (*B*. *abortus* 2308), and 7.19 *(B*. *suis* 1330) Log_10_ CFUs/spleen.

**Table 1:** *Brucella* presumptive tests.

*Brucella* strain	Gram Stain	Rough/Smooth	Agglutination with Serum	Acriflavin	Oxidase	Catalase	Thionin	Basic Fuchsin
*B*. *abortus* 2308	−	smooth	+	−	+	+	+	−
*B*. *suis* 1330	−	smooth	+	−	+	+	+	−
*B*. *neotomae* ATCC 23459	−	smooth	+	−	−	+	+	−
*B*. *neotomae* Passaged 2	−	smooth	+	−	−	+	+	−

Each passage of *B*. *neotomae* was characterized through various biochemical and microbiology tests to determine each passages similarity to the parent strain while distinguishing them from other *Brucella* spp.

### *B*. *neotomae* ATCC 23459 and *B*. *abortus* 2308 in naïve murine macrophages

**Fig 2:**
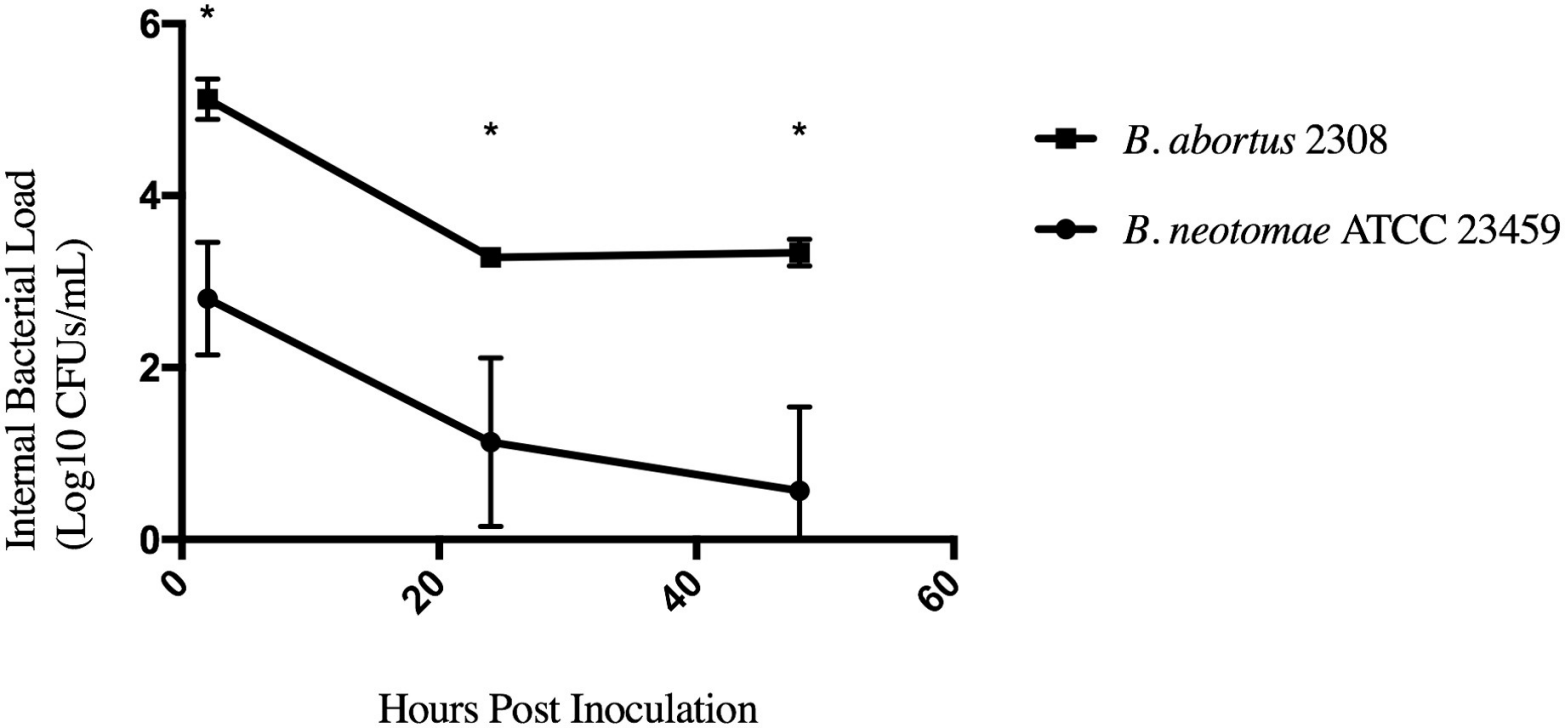
Intracellular survival of *B*. *abortus* 2308 versus *B*. *neotomae* ATCC 23459 in naïve murine macrophages. The naïve murine peritoneal macrophages were infected as described above and represent the internal bacterial CFUs of BN and *B*. *abortus* 2308 over 48hrs. Error bars represent the standard error, and * represent species tested that differ from BN, *p* < 0.05. Each indicator corresponds to the mean of three replicate wells from one assay.

BN and virulent *B*. *abortus* 2308 were incubated with sera from mice that had been infected with BN. The effects of incubation with sera from mice infected with BN or virulent *B*. *abortus* 2308 was determined (S4 Fig) as it has been documented that BN antibodies cross react with *B*. *abortus* 2308 antibodies [10]. The recovered Log_10_ CFUs/mL of intracellular BN at each time point (2 hours, 6 hours, 24 hours, and 48 hours post infection) over 48hrs was significantly lower than the Log_10_ CFUs/mL of intracellular *B*. *abortus* 2308 recovered over 48 hours (Fig 2). It is important to note that no bacterial growth was observed from all plated infected cell culture supernatant for all the described gentamicin protection assays.

### Entrance and survival of *Brucella* strains compared to *B*. *neotomae* ATCC 23459 in epithelial cells

**Fig 3:**
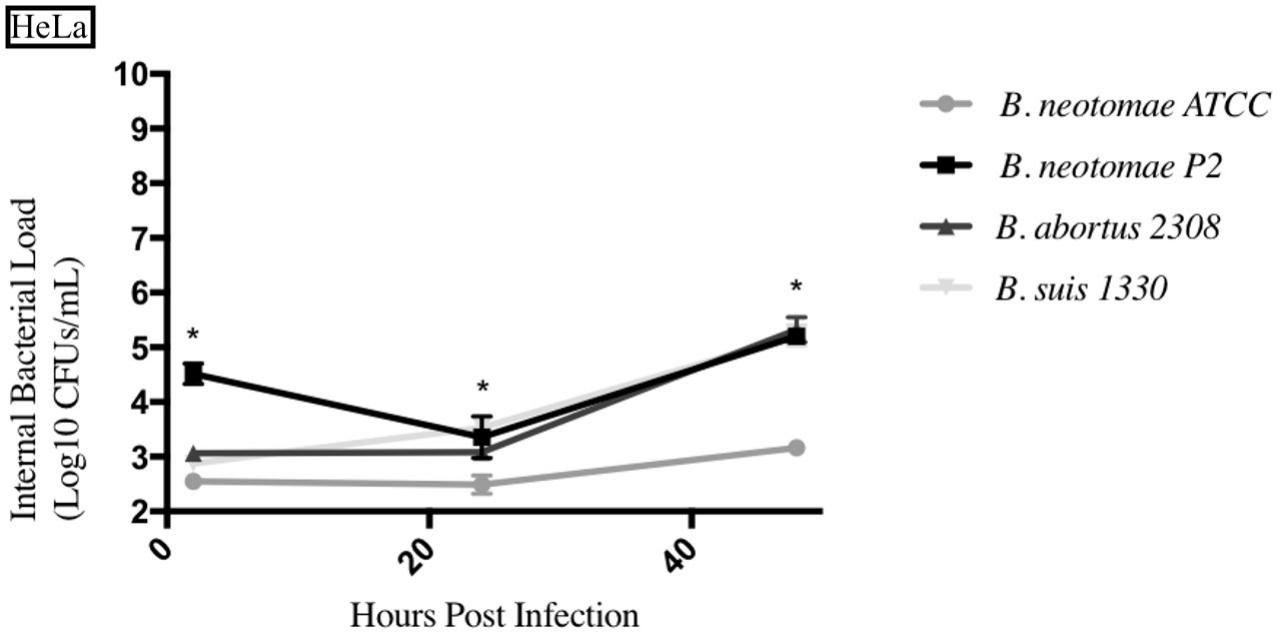
Intracellular survival of *Brucella* species in the human epithelial cell line (HeLa). Intracellular *Brucella* spp. bacterial CFUs were determined over 48hrs in HeLa as described. Error bars represent the standard deviation of the triplicates from a single experiment. * represents when intracellular CFUs from more than one species tested statistically differs from BN with any *p* value lower than 0.05.

**Fig 4:**
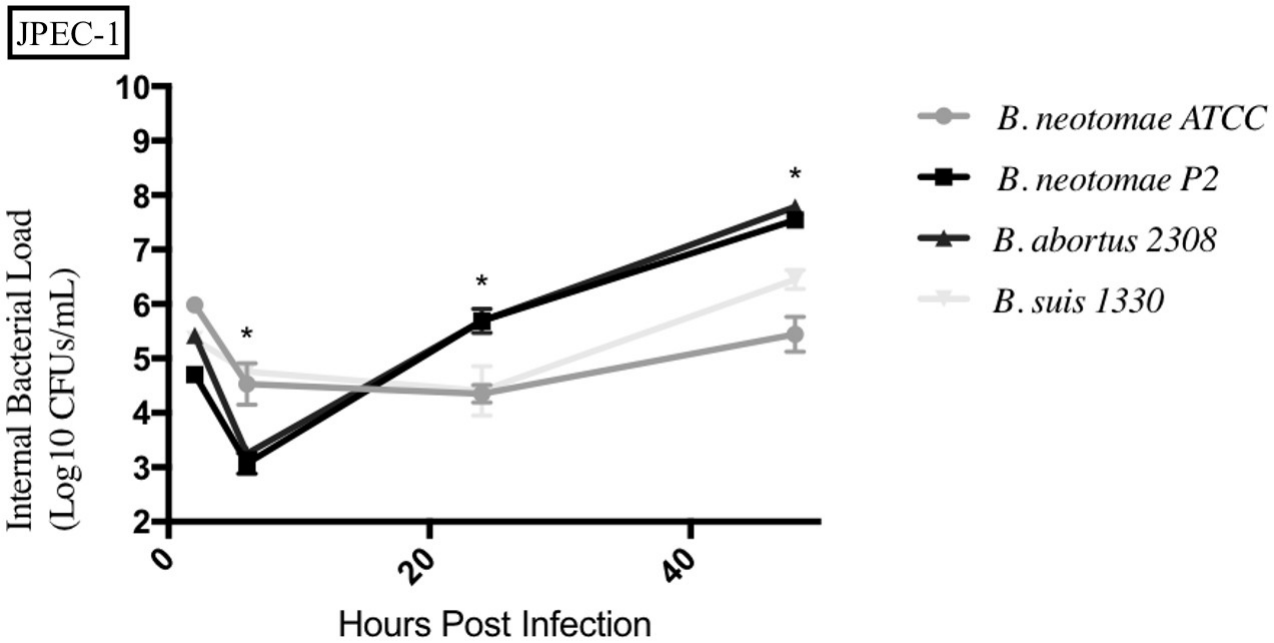
Intracellular survival of *Brucella* species in the swine jejunum epithelial cell line (JPEC-1). Intracellular *Brucella* spp. bacterial CFUs were determined over 48hrs in JPEC-1 as described. Error bars represent the standard deviation of the triplicates from a single experiment. * represents when intracellular CFUs from more than one species tested statistically differs from BN with any *p* value lower than 0.05.

**Fig 5:**
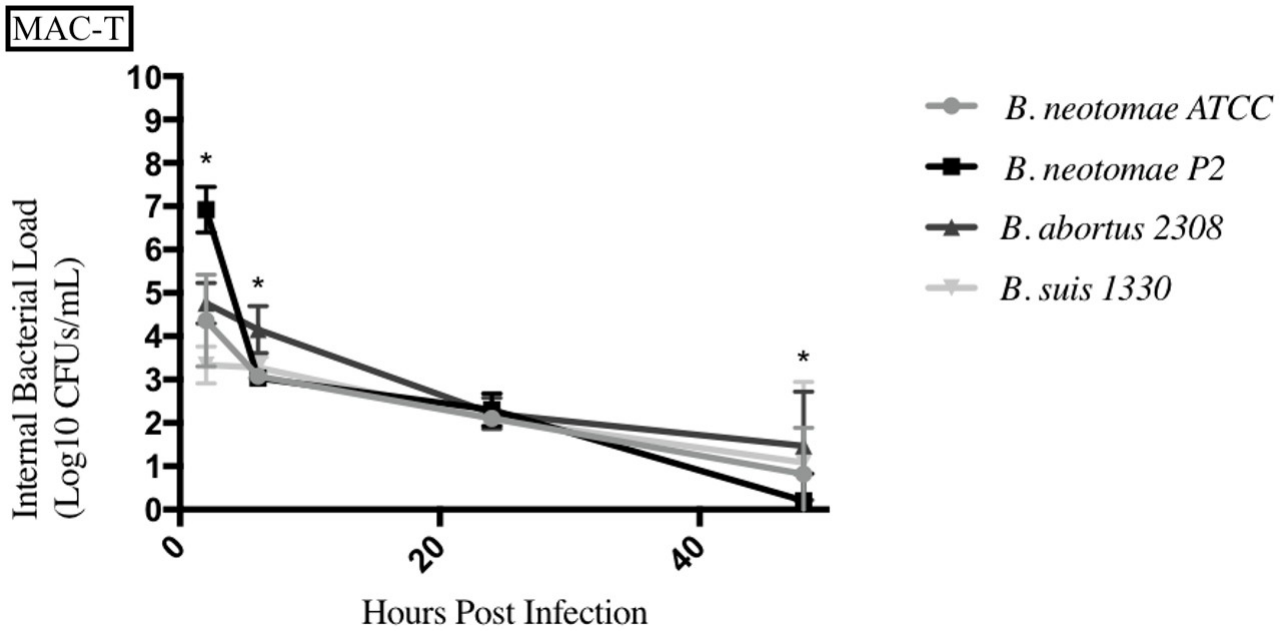
Intracellular survival of *Brucella* species in the bovine mammary cell line (MAC-T). Intracellular *Brucella* spp. bacterial CFUs were determined over 48hrs in MAC-T as described. Error bars represent the standard deviation of the triplicates from a single experiment. * represents when intracellular CFUs from more than one species tested statistically differs from BN with any *p* value lower than 0.05.

For each time point, the intracellular bacterial CFUs (Log_10_ CFUs/mL) of *B*. *neotomae* ATCC 23459 passage 2 (BNP2), *B*. *suis* 1330, and *B*. *abortus* 2308 were statistically compared to that of *B*. *neotomae* ATCC 23459 (BN). *B*. *suis* 1330 and *B*. *abortus* 2308 are known pathogenic strains. The results from each epithelial cell line tested can be seen in Figs 3–5, and a summary of all the statistical results can be seen in S1–S4 Tables.

BNP2 had significantly higher intracellular bacterial CFUs in both the human epithelial cell line (HeLa) and the swine jejunum epithelial cell line (JPEC-1), with only statistical differences seen at the 24hrs and 48hrs time points in JPEC-1. BNP2 generally had significantly similar intracellular bacterial CFUs in the bovine mammary cell line (MAC-T), compared to BN. *B*. *suis* 1330 also had significantly higher intracellular CFUs in HeLa cells, while only having significantly higher CFUs at 6hrs in MAC-T cells and 48hrs in JPEC-1 cells. *B*. *suis* 1330 also had lower intracellular CFUs at the 2hrs time point in MAC-T cells compared to BN. *B*. *abortus* 2308 generally had significantly higher intracellular CFUs in all three epithelial cell lines (HeLa, JPEC-1, and MAC-T) tested compared to BN.

### Uptake and survival of *Brucella* strains compared to *B*. *neotomae* ATCC 23459 in phagocytic cells

**Fig 6:**
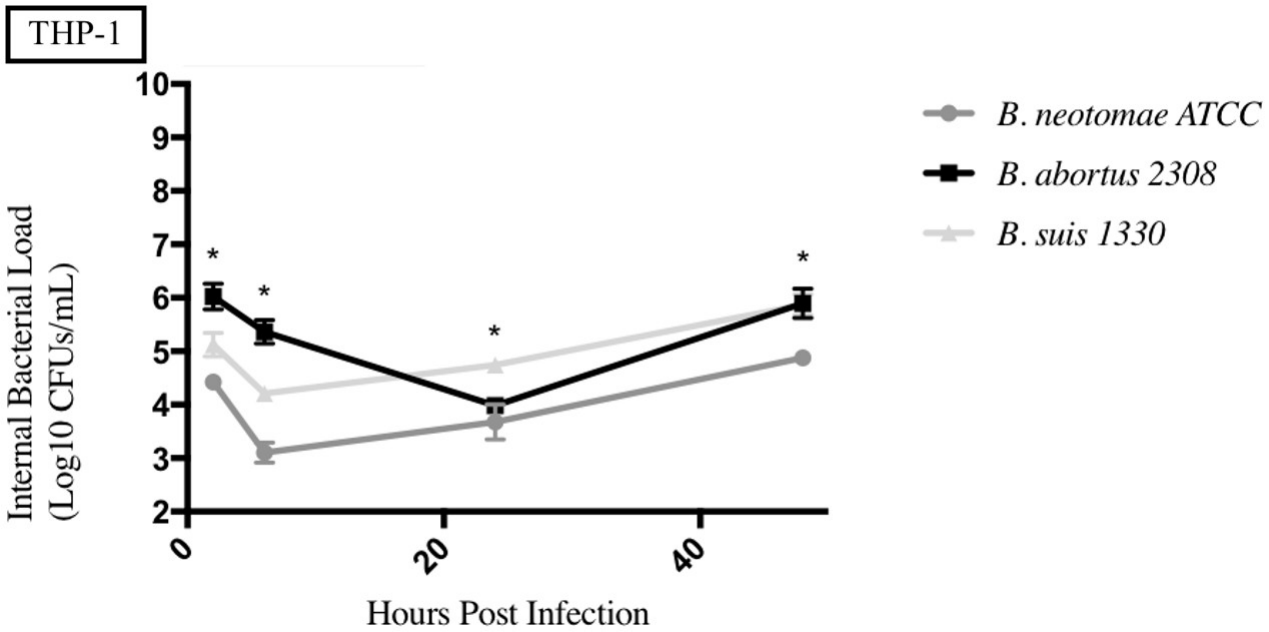
Intracellular survival of *Brucella* species in the human macrophage cell line (THP-1). Intracellular *Brucella* spp. CFUs were determined over 48hrs in THP-1 as described. Error bars represent the standard deviation of the triplicates from a single experiment. * represents when intracellular CFUs from more than one species tested statistically differs from BN with any *p* value lower than 0.05.

**Fig 7:**
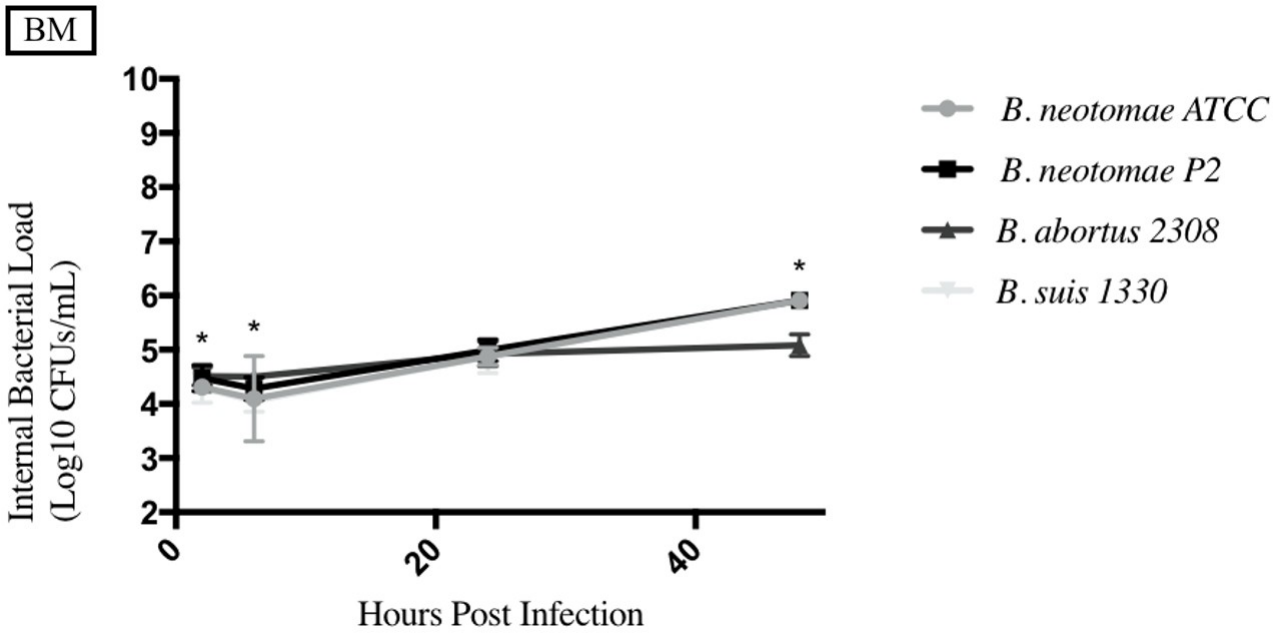
Intracellular survival of *Brucella* species in the bovine macrophage cell line (BM). Intracellular *Brucella* spp. CFUs were determined over 48hrs in BM as described. Error bars represent the standard deviation of the triplicates from a single experiment. * represents when intracellular CFUs from more than one species tested statistically differs from BN with any *p* value lower than 0.05.

**Fig 8:**
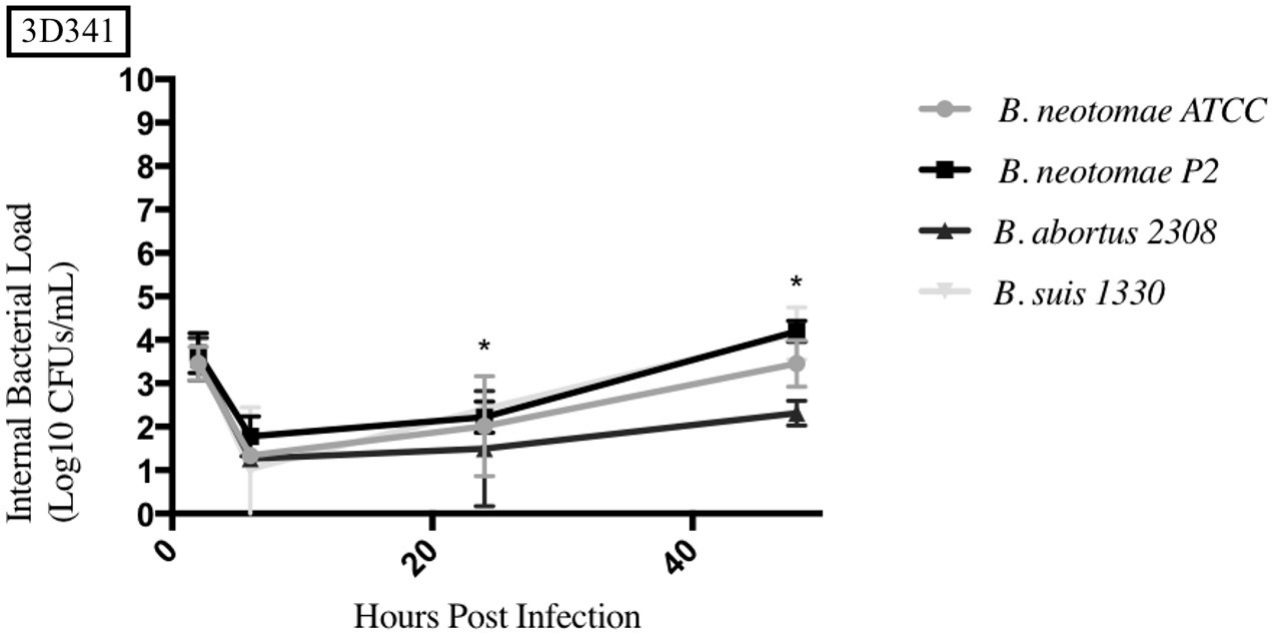
Intracellular survival of *Brucella* species in the swine monocyte cell line (3D4/31). Intracellular *Brucella* spp. CFUs were determined over 48hrs in 3D4/31 as described. Error bars represent the standard deviation of the triplicates from a single experiment. * represents when intracellular CFUs from more than one species tested statistically differs from BN with any *p* value lower than 0.05.

**Fig 9:**
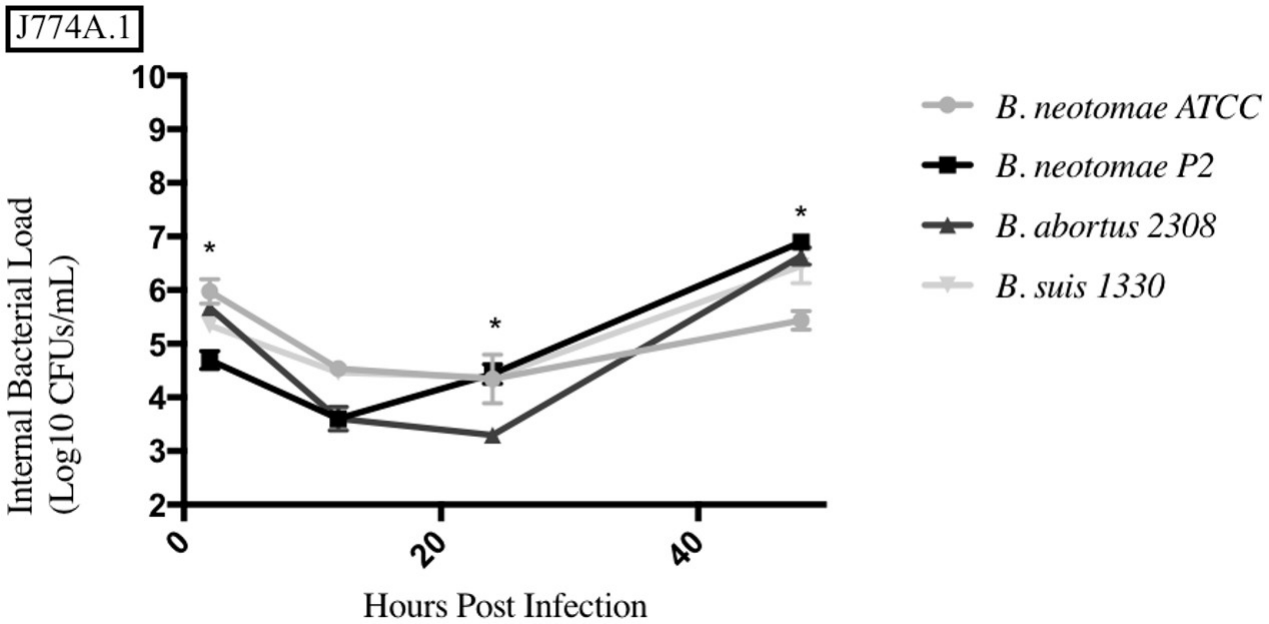
Intracellular survival of *Brucella* species in the murine macrophage cell line (J774A.1). Intracellular *Brucella* spp. CFUs were determined over 48hrs in J774A.1 as described. Error bars represent the standard error of the triplicates from a two seperate experiments. * represents when intracellular CFUs from more than one species tested statistically differs from BN with any *p* value lower than 0.05.

**Fig 10:**
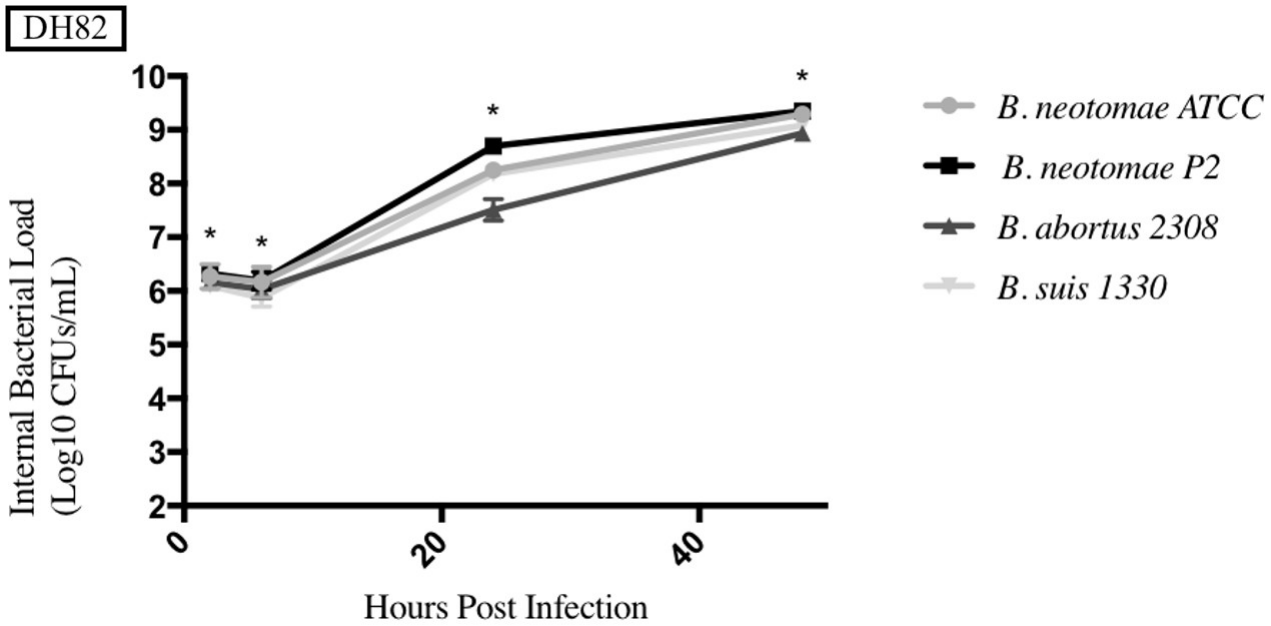
Intracellular survival of *Brucella* species in the dog macrophage cell line (DH82). Intracellular *Brucella* spp. CFUs were determined over 48hrs in DH82 as described. Error bars represent the standard deviation of the triplicates from a single experiment. * represents when intracellular CFUs from more than one species tested statistically differs from BN with any *p* value lower than 0.05.

The results from each phagocytic cell line tested can be seen in Figs 6–10, and a summary of all the statistical results can be seen in S1–S4 Tables. BNP2 was not tested in the human macrophage cell line (THP-1), but had statistically higher intracellular bacterial CFUs to BN in the bovine macrophage cell line (BM) at 2 and 6hrs, swine monocyte cell line (3D4/31) at 48hrs, murine macrophage cell line (J774A.1) at 48hrs, and dog macrophage cell line (DH82) at 48hrs. There was only one time point that BNP2 intracellular CFUs were significantly lower than BN; at 2hrs post infection in J774A.1 cells. *B*. *abortus* 2308 had significantly higher intracellular CFUs in THP-1 and BM cell lines. In DH82 and 3D4/31, *B*. *abortus* 2308 generally had lower intracellular CFUs compared to BN, while intracellular CFUs were significantly higher (48hrs) and lower (24hrs) at one time point in the J774A.1 cell line. Intracellular CFUs of *B*. *suis* 1330 were statistically similar to those of BN in THP-1, DH82, and BM cell lines, with a few exceptions. The 24hrs and 48hrs intracellular bacterial CFUs in 3D4/31 cells were significantly higher than BN’s, as well as the 48hrs time point in J774A.1 cells. The intracellular CFUs at the 2hrs time point in J774A.1 and 48hrs time point post infection in DH82 cell lines were significantly lower than BN intracellular CFUs.

## Discussion

Cell culture is widely used as a representative model for microorganism’s infectivity and is especially important in helping predict the range of pathogenicity of atypical *Brucella* species [16, 35–37]. Invasion and replication in epithelial cells and macrophages are vital in *Brucella* infections, although they are just one aspect to virulence [16, 37]. Other aspects important to virulence are the type IV secretion system and the LPS O-side chain [11, 13, 23, 35, 36]. Current literature supports that *B*. *neotomae* possesses these virulence factors [4, 16, 35]. *B*. *neotomae* has also been shown to have type IV dependent intracellular survival and a similar late endoplasmic reticulum associated phagosome stage to that of other virulent *Brucella* spp. [11]. Ultimately, it was expected to see *B*. *neotomae* strains have the ability to survive within phagocytic and epithelial cell lines in a similar manor to other virulent species, especially with current literature showing that it can infect humans, swine, rodents, and bison [11, 12, 14, 20, 24, 37]. Our intracellular invasion and survival profiles of *B*. *neotomae* add to the evidence in current literature warranting further characterization of its pathogenic ability. Our data aligns with current literature and extends the known intracellular uptake and survival profiles of the *Brucella* species to include a breadth of cell lines [38–46].

The mucous membranes of the respiratory and digestive tracts are the most common sites of entry for *Brucella* species, with epithelial cell invasion a crucial initial stage of infection [37, 39, 42, 43]. We show that *B*. *neotomae* can persist in cultured epithelial cells, although the other strains tested generally maintained higher intracellular CFUs. Mammary tissue has been considered a known route of infection for *Brucella*, but a major reduction in intracellular bacterial numbers was observed in the bovine mammary epithelial cells (MAC-T). More in depth experiments with various strains and types of mammary tissues need to be conducted to confirm this observation [35]. It would also be worth while to determine other tissue types that *B*. *neotomae* can be isolated from in various species and whether those infected tissues can be a source of infection to other potential hosts. It is important to note that current literature has shown MAC-T cells have the ability to clear intracellular organisms, which could possibly explain this observation [35, 45].

After crossing the epithelial cell border, phagocytic cells have been shown to be an important site for *Brucella* intracellular replication within host tissues [33, 37, 42, 45]. Although internalized in low numbers, enough cells are able to activate virulence genes and establish a replication niche to produce chronic infection [33, 42, 45]. Our data shows that *Brucella* 2308, *B*. *suis* 1330, and BNP2 generally had higher intracellular bacterial CFUs in phagocytic cell lines compared to BN.

*B*. *abortus* 2308 and BN exhibited a downward trend in naïve murine peritoneal macrophages, which brings into question the validity of the common practice of testing *Brucella* species in cultured cell lines. Further biological replicates need to be done along with determining the intracellular uptake and survival profiles in non-immortalized cell culture to better understand this phenomenon. It is of importance to take into account that survival in phagocytic cells can be negatively affected by opsonization [35]. This may contribute to the naïve murine peritoneal macrophage intracellular profiles observed as non-opsonized *Brucella* infection of naïve macrophages in current literature shows higher intracellular replication [12, 13, 35, 43, 44]. *B*. *neotomae* antibodies are grouped under *B*. *abortus*-*B*. *suis*, but no studies have been done to determine their effects on BN intracellular survival [10–12, 18, 25, 46]. We have shown that opsonization with different mouse sera can significantly impact initial uptake into the cell and further investigation needs to be done to understand this effect on intracellular uptake and survival.

The limitations of cell culture are important to take into account when interpreting cell culture data like ours. Cell culture can differ genetically and phenotypically from primary cell culture, among other limitations including a lack of biological stratification and influence that the host provides. Although this is important to take into consideration, there is a large body of evidence in current literature that depicts the intracellular profiles of *Brucella* and it is common practice to characterize them in immortalized cell culture [23, 35, 39, 44, 47, 48–51].

Furthermore, it is of interest to highlight the general significantly higher intracellular bacterial CFUs exhibited by BNP2 compared to the parent strain BN. As stated, it has been shown that passage through rodents produces variations in virulence of *B*. *neotomae*. It was important to compare the classical virulent and passaged strains to the *B*. *neotomae* ATCC strain to observe any differences. There were some significant differences between the parent and passaged *B*. *neotomae* strains at various time points, but more experiments need to be completed to better understand this. In particular, BNP2 was statistically higher than BN in HeLa, JPEC-1, and most macrophage cell cultures [20].

In conclusion, this work shows that *B*. *neotomae* possesses the ability to invade and survive in cultured phagocytic and epithelial cells derived from a wide array of species. In general, *B*. *abortus* 2308, *B*. *suis* 1330, and *B*. *neotomae* passage 2 had statistically higher intracellular bacterial CFUs compared to *B*. *neotomae* ATCC 23459. Our data, along with the current literature warrants that the currently accepted thought that *B*. *neotomae* lacks pathogenic ability needs to be investigated [7, 9, 25, 47]. Future research is needed to better understand *B*. *neotomae*’s ability (genotypically and phenotypically) to infect a variety of hosts, the tissues in which it replicates, the infectious dose and host immune status required to cause disease, the routes of exposure to cause infection, the host’s immunological response to *B*. *neotomae*, and the affects of passaging on the species.

## Supporting information

S1 Fig *B*. *neotomae* ATCC 23459 (BN) on TSA containing thionin. BN was tested for growth on TSA containing thionin dye as described. Single colonies can be visualized.
(DOCX)

S2 Fig *B*. *neotomae* ATCC 23459 (BN) on TSA containing basic fuchsin dye. BN was tested for growth on TSA containing basic fuchsin dye as described. There was no growth.
(DOCX)

S3 Fig *B*. *neotomae* ATCC 23459 passage 2 (BNP2) on TSA containing dye. BNP2 was tested for growth on TSA containing thionin and basic fuchsin dye as described. Growth can be visualized on thionin.
(DOCX)

S4 Fig Opsonized *Brucella* species in naïve macrophages. Each bar represents the Log_10_CFUs/mL of intracellular *B*. *abortus* 2308 or BN recovered from naïve murine macrophages 2hrs post-infection, as described above. Error bars represent the standard error, and * represent *p* < 0.05. Each indicator corresponds to the mean of three replicates from one assay.
(DOCX)

S1 Table Bacterial load (Log_10_CFUs/mL) of suspensions used to challenge cell lines. (DOCX)

S2 Table Significant differences between BNP2 compared to BN. (+ = statistically higher internal bacterial loads, − = statistically lower internal bacterial loads, 0 = no statistical difference).
(DOCX)

S3 Table Significant differences between *B*. *abortus* 2308 compared to BN. (+ = statistically higher internal bacterial loads, − = statistically lower internal bacterial loads, 0 = no statistical difference).
(DOCX)

S4 Table Significant differences between *B*. *suis* 1330 compared to BN. (+ = statistically higher internal bacterial loads, − = statistically lower internal bacterial loads, 0 = no statistical difference).
(DOCX)
