# Supplementary material for: Intracellular invasion and survival of Brucella neotomae, another possible zoonotic Brucella species
Source: PLoS One. 2019 Apr 3;14(4):e0213601. doi: 10.1371/journal.pone.0213601 (PMC6447175; doi:10.1371/journal.pone.0213601)
Supplement: S1 Fig — BN was tested for growth on TSA containing thionin dye as described. Single colonies can be visualized. (DOCX) [file pone.0213601.s001.docx]

S1 Fig. *B. neotomae* ATCC 23459 (BN) on TSA containing thionin.


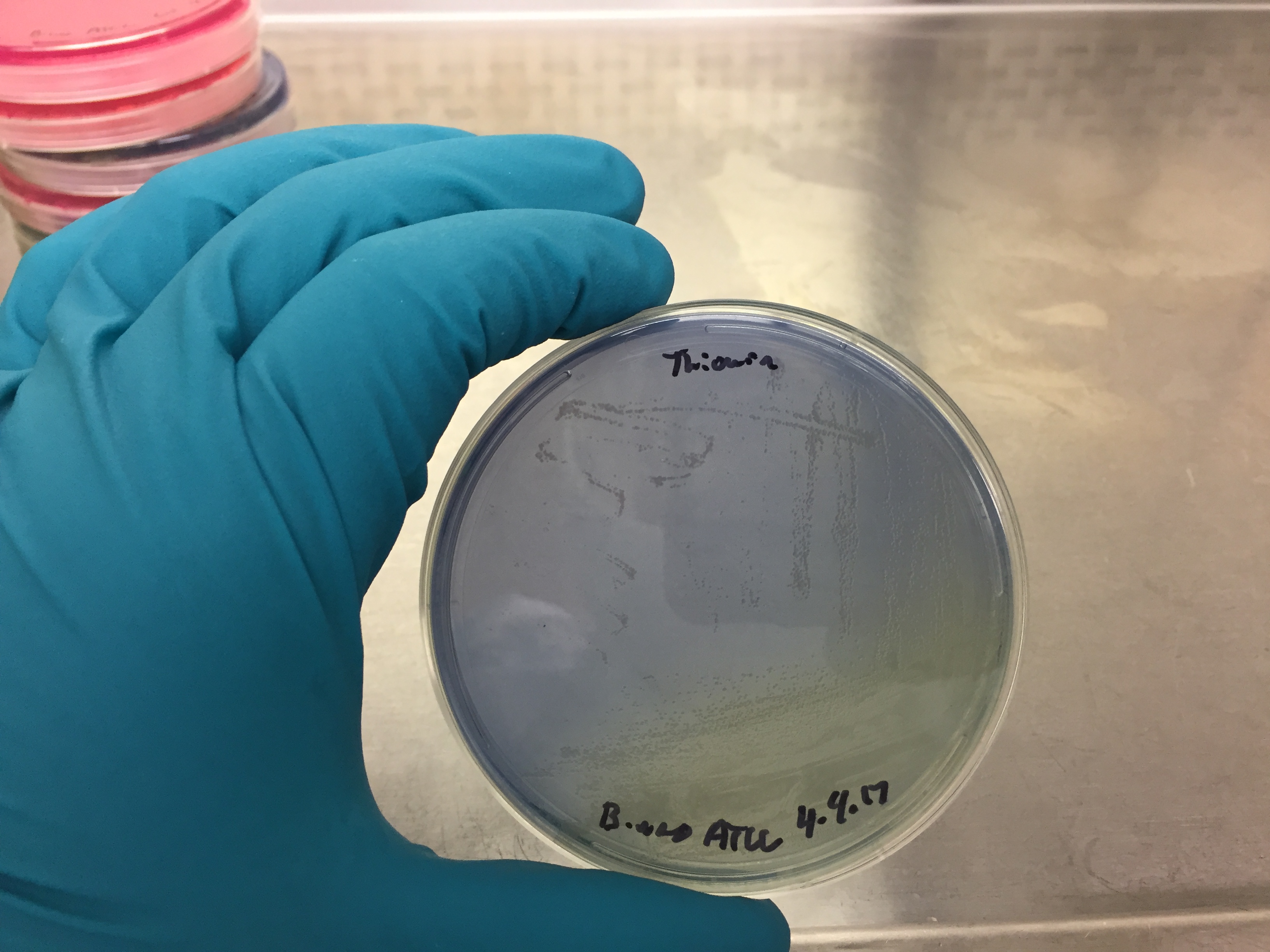


BN was tested for growth on TSA containing thionin dye as described. Single colonies can be visualized.
