# Supplementary material for: Intracellular invasion and survival of Brucella neotomae, another possible zoonotic Brucella species
Source: PLoS One. 2019 Apr 3;14(4):e0213601. doi: 10.1371/journal.pone.0213601 (PMC6447175; doi:10.1371/journal.pone.0213601)
Supplement: S3 Fig — BNP2 was tested for growth on TSA containing thionin and basic fuchsin dye as described. Growth can be visualized on thionin. (DOCX) [file pone.0213601.s003.docx]

S3 Fig. *B. neotomae* ATCC 23459 passage 2 (BNP2) on TSA containing dye.


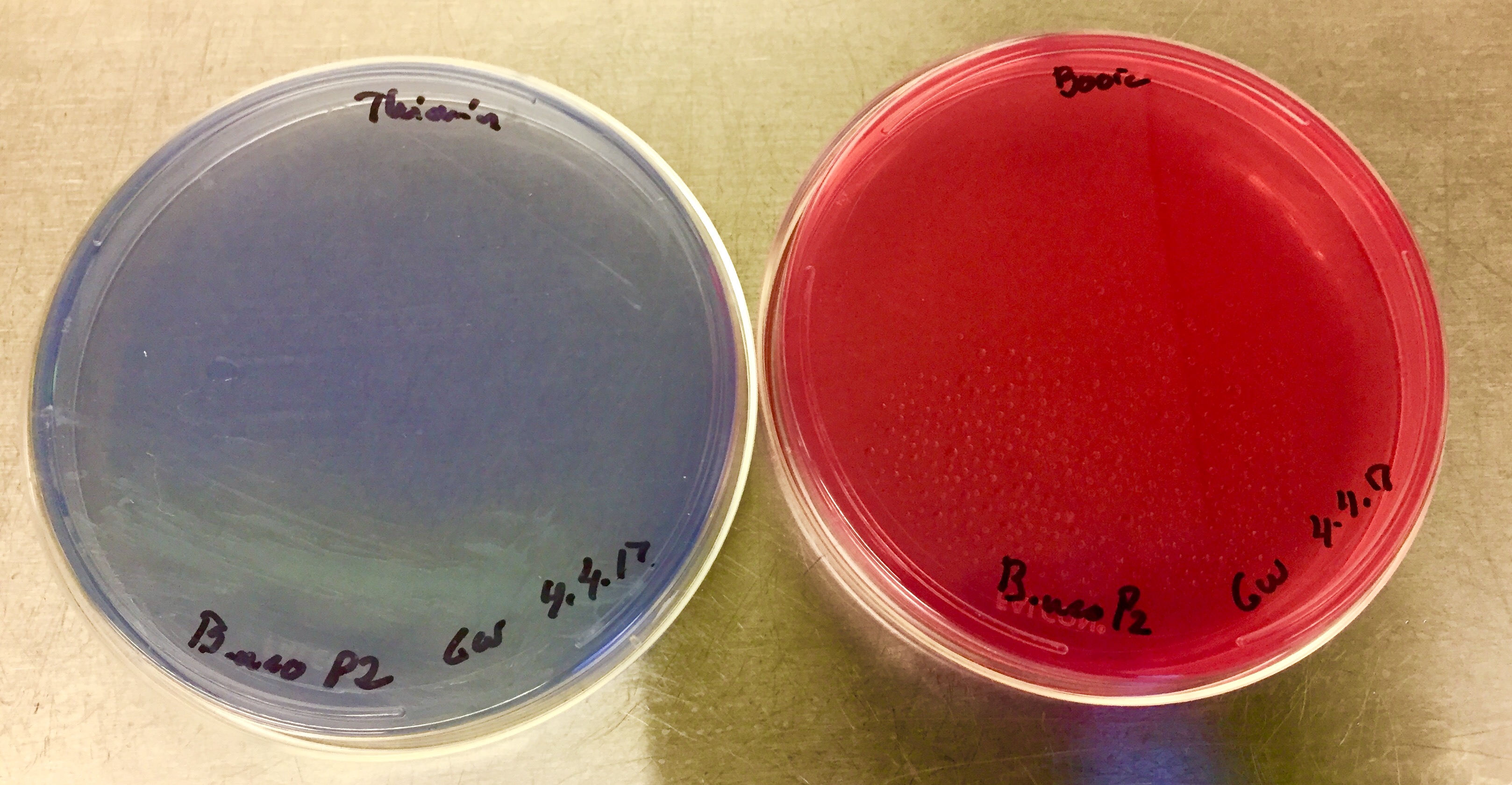


BNP2 was tested for growth on TSA containing thionin and basic fuchsin dye as described. Growth can be visualized on thionin.
