# Supplementary material for: Intracellular invasion and survival of Brucella neotomae, another possible zoonotic Brucella species
Source: PLoS One. 2019 Apr 3;14(4):e0213601. doi: 10.1371/journal.pone.0213601 (PMC6447175; doi:10.1371/journal.pone.0213601)
Supplement: S4 Fig — Each bar represents the Log10CFUs/mL of intracellular B. abortus 2308 or BN recovered from naïve murine macrophages 2hrs post-infection, as described above. Error bars represent the standard error, and * represent p < 0.05. Each indicator corresponds to the mean of three replicates from one assay. (DOCX) [file pone.0213601.s004.docx]

S4 Fig. Opsonized *Brucella* species in naïve macrophages.


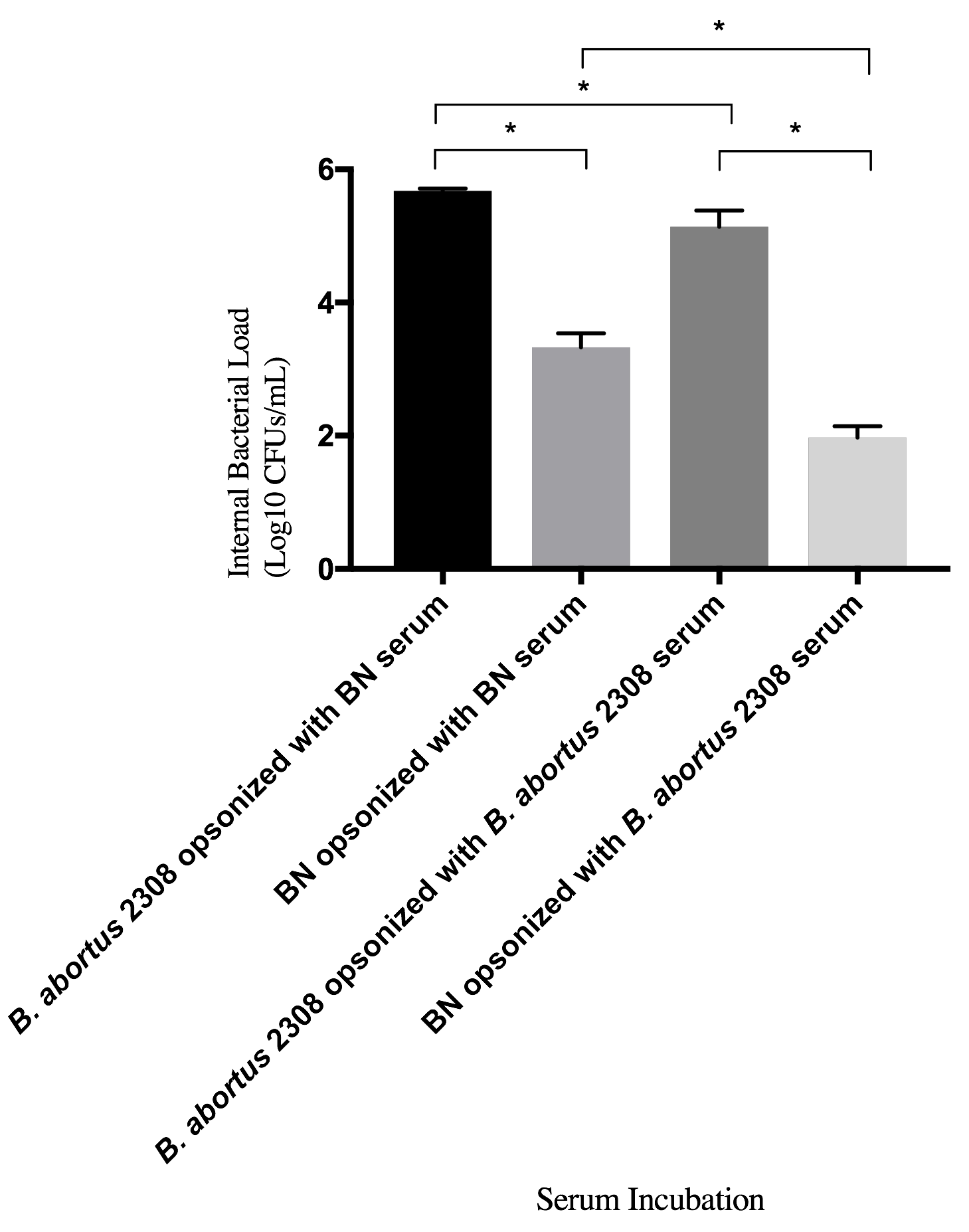


Each bar represents the Log_10_CFUs/mL of intracellular *B. abortus* 2308 or BN recovered from naïve murine macrophages 2hrs post-infection, as described above. Error bars represent the standard error, and * represent *p* < 0.05. Each indicator corresponds to the mean of three replicates from one assay.
