# Supplementary material for: Intracellular invasion and survival of Brucella neotomae, another possible zoonotic Brucella species
Source: PLoS One. 2019 Apr 3;14(4):e0213601. doi: 10.1371/journal.pone.0213601 (PMC6447175; doi:10.1371/journal.pone.0213601)
Supplement: S1 Table — (DOCX) [file pone.0213601.s005.docx]

S1 Table. Bacterial load (Log_10_CFUs/mL) of suspensions used to challenge cell lines.

|  | J774A.1 | DH82 | THP-1 | HeLa | BM | MAC-T | 3D4/31 | JPEC-1 |
| --- | --- | --- | --- | --- | --- | --- | --- | --- |
| *B. neotomae* ATCC 23459 (BN) | 8.13 | 8.13 | 8.65 | 8.13 | 8.13 | 8.0 | 8.13 | 8.11 |
| *B. neotomae* ATCC 23459 passage 2 (BNP2) | 7.85 | 7.85 | 8.02 | 7.85 | 7.85 | 8.01 | 7.55 | 8.05 |
| *B. abortus* 2308 | 7.53 | 7.53 | 7.88 | 7.53 | 7.53 | 7.98 | 7.53 | 7.85 |
| *B. suis* 1330 | 8.01 | 8.01 | 7.59 | 8.01 | 8.01 | 7.88 | 8.01 | 7.87 |
