# Supplementary material for: Intracellular invasion and survival of Brucella neotomae, another possible zoonotic Brucella species
Source: PLoS One. 2019 Apr 3;14(4):e0213601. doi: 10.1371/journal.pone.0213601 (PMC6447175; doi:10.1371/journal.pone.0213601)
Supplement: S2 Table — (+ = statistically higher internal bacterial loads, − = statistically lower internal bacterial loads, 0 = no statistical difference). (DOCX) [file pone.0213601.s006.docx]

S2 Table. Significant differences between BNP2 compared to BN.

|  | THP-1 | HeLa | J774A.1 | DH82 | 3D4/31 | JPEC-1 | BM | MAC-T |
| --- | --- | --- | --- | --- | --- | --- | --- | --- |
| 2hrs | Not tested | + | − | 0 | 0 | 0 | + | + |
| 6hrs | Not tested | + | 0 | 0 | 0 | 0 | + | − |
| 24hrs | Not tested | + | 0 | + | 0 | + | 0 | 0 |
| 48hrs | Not tested | + | + | 0 | + | + | 0 | − |

(+ = statistically higher internal bacterial loads, − = statistically lower internal bacterial loads, 0 = no statistical difference)
